# Choice of a family planning outlet in urban areas: The role of distance and quality of services in Kenya and Uganda

**DOI:** 10.3389/fgwh.2023.1117849

**Published:** 2023-03-30

**Authors:** Jennifer Winston, Lisa M. Calhoun, David Guilkey, Peter M. Macharia, Ilene S. Speizer

**Affiliations:** ^1^ Carolina Population Center, University of North Carolina at Chapel Hill, Chapel Hill, NC, United States; ^2^ Department of Economics, University of North Carolina at Chapel Hill, Chapel Hill, NC, United States; ^3^ Centre for Health Informatics, Computing, and Statistics, Lancaster Medical School, Lancaster University, Lancaster, United Kingdom; ^4^ Population Health Unit, Kenya Medical Research Institute – Wellcome Trust Research Programme, Nairobi, Kenya; ^5^ Department of Maternal and Child Health, Gillings School of Public Health, University of North Carolina at Chapel Hill, Chapel Hill, NC, United States

**Keywords:** willingness to travel, facility choice, quality of care, family planning, Kenya, Uganda

## Abstract

**Introduction:**

Quality of care and physical access to health facilities affect facility choice for family planning (FP). These factors may disproportionately impact young contraceptive users. Understanding which components of service quality drive facility choice among contraceptive users of all ages can inform strategies to strengthen FP programming for all potential users of FP.

**Methods:**

This study uses data from Population Services International's Consumer's Market for Family Planning (CM4FP) project, to examine drivers of facility choice among female FP users. The data collected from female contraceptive users, the outlet where they obtained their contraceptive method, and the complete set of alternative outlets in select urban areas of Kenya and Uganda were used. We use a mixed logit model, with inverse probability weights to correct for selection into categories of nonuse and missing facility data. We consider results separately for youth (18–24) and women aged 25–49 in both countries.

**Results:**

We find that in both countries and across age groups, users were willing to travel further to public outlets and to outlets offering more methods. Other outlet attributes, including signage, pharmacy, stockouts, and provider training, were important to women in certain age groups or country.

**Discussion:**

These results shed light on what components of service quality drive outlet choice among young and older users and can inform strategies to strengthen FP programming for all potential users of FP in urban settings.

## 1. Introduction

Recent revisions of Judith Bruce's 1990 seminal framework on quality of care (1) emphasize the distinction between structural quality, including service delivery point readiness, and process quality, which is focused on the interpersonal relations between providers and clients (2–4). Structural quality of care components, which is the focus of this paper, such as method availability and stock-outs, wait time, or facility opening hours affect service users of all ages, yet these factors and several others disproportionately impact young contraceptive users (5, 6). Young contraceptive users (aged 15–24) prefer facilities with flexible hours, short wait times, and convenient service locations (7, 8). Given that young people face unique barriers to accessing the contraceptive method of their choice, specific components of structural service quality may drive young contraceptive users to select health facilities based on these quality related characteristics.

Evidence from rural settings has shown that further distance and increased travel time is associated with less facility use for family planning (FP) and childbirth (9, 10). One limitation of much of the extant literature examining the effect of distance and quality on service use is that for these analyses respondents are frequently linked to the nearest facility to their home or an aggregate measure of features of proximate facilities is created; neither of these approaches examine where respondents actually seek FP services (11). A systematic review of household and facility linking strategies used in reproductive, maternal, newborn, and child health (RMNCH) research found that only 14% (8 out of 59) of the studies that were included in the review directly linked households and facilities where care was obtained (11). This is problematic because studies from urban areas, which are inherently more complex than rural areas, have highlighted that women often bypass the nearest facility (12–14) to seek services that are perceived to be of higher quality, among other reasons (12–15).

In Kenya and Uganda, the sites of this study, accessibility to and bypassing of health facilities for RMNCH services has been a topic of interest. A 2018 study from Escamilla and colleagues (15) found that female contraceptive users residing in urban sites in Kenya commonly bypassed the nearest facility to their home to seek higher quality FP services and to visit public facilities. More recent evidence from urban sites in Kenya found that while about 20% of contraceptive users went to the closest outlet for services, about half of users went to the closest outlet conditional on the outlet offering the FP method used most recently by the respondent (16). Similar evidence on facility bypassing for FP services is sparser in Uganda, but there is evidence of women bypassing the nearest facility to their home for childbirth (17, 18).

Although earlier studies have identified the role of facility quality characteristics on facility choice for contraception among women of all ages (12–14), less is known about this relationship for young women and how it compares to women over the age of 25. Using a unique dataset that links female contraceptive users to all health outlets within their geographic area, this study explores the association between structural facility quality characteristics and women's decision of where to obtain their contraceptive method of choice in urban sites in Kenya and Uganda. In addition, because young women often face unique barriers to FP services, we determine if different service quality factors are associated with choice of outlet among women aged 18–24 compared to women aged 25–49 and if these factors differ across countries. While adolescents aged 10–17 likely face additional barriers to FP access (19), data were unavailable for this age group. For this analysis, we examine differences in FP access between adolescent and young women aged 18–24 and their older peers aged 25–49 to determine if the younger age group faces a greater burden to access and use. Understanding which components of service quality drive facility choice among different age groups can inform strategies to strengthen FP programming for all potential users of FP.

## 2. Methods

### 2.1. Context: Kenya and Uganda

As of 2021, nearly half (46.2%) of all women aged 15–49 in Kenya were using a modern contraceptive method (female and male sterilization, implant, IUD, Injectables, oral pills, emergency contraceptives, male and female condoms, and standard days method), although use was lower among adolescents and youth aged 15–24 (29.5%) than among women aged 25–49 (57.3%). Among users of modern methods, women aged 25–49 were more likely to use long-acting methods (44.9%) compared to women aged 15–24 (33.2%) and injectables (36.7% of women aged 25–49 compared to 29.3% of women aged 15–24), while adolescents and youth were more likely to use other, usually shorter acting, modern methods (37.5% and 18.4%, respectively) (20). Most women (73.8%), no matter the age group, obtained their method from a governmental facility (20). In the same year, modern contraceptive use in Uganda was lower (34.0%), with only 23.6% of all women aged 15–24 using a modern method. Similar to Kenya, among users of modern methods in Uganda, women aged 25–49 were more likely than women aged 15–24 to use long-acting methods (40.3% compared to 35.5%) or injectables (23.5% vs. 20.8%), while adolescents and youth were more likely than women aged 25–49 to use other modern methods (43.7% compared to 36.2%) (21). Also similar to Kenya, most women in Uganda (66.9%) obtained their method from a governmental facility (21).

Both countries have made FP2030 commitments to increase modern contraceptive use and to better meet adolescent FP needs. In Kenya, the government has committed to increase modern contraceptive prevalence to 66% by 2030 and to reduce teen pregnancy to 10% by 2025 (22). In its FP2030 commitments, the Government of Uganda committed to increase modern contraceptive use to 39.6% by 2025, as well as to annually allocate at least 10% of Maternal and Child Health resources to adolescent health needs (23).

### 2.2. Data

Data for this study come from Population Services International's Consumer's Market for Family Planning (CM4FP) project which are publicly available (24). In 2019–2020, data were collected from women in households and outlets in 12 sites across three countries: Kenya, Uganda, and Nigeria (4 sites per country, primarily urban). This paper utilizes data from Kenya and Uganda because they had a greater number of respondents, including youth contraceptive users, directly linked to an outlet.

In Kenya, data were collected in Nairobi County (large-urban), Nakuru County (medium-urban), Kilifi County (small-urban), and Migori County (peri-urban). In Uganda, data were collected in Kampala district (large-urban), Mbarara district (medium-urban), Gulu district (small-urban), and Soroti district (rural). Sites were selected based on programmatic and funder priorities while representing a continuum of geographic areas. While selection was not intended to be representative at a population level, these data provide unique insight into individual level decision making in primarily urban contexts of Kenya and Uganda by linking women to a full census of FP outlets within their supply environment (25).

Outer rings based on contiguous administrative wards (Kenya) and parishes (Uganda) were drawn at each site at a size to reach an estimated sample of 600 outlets offering contraception in each country. The outer rings did not include complete cities or counties. In each outer ring, an initial census of all service delivery points offering more than just condoms was undertaken. Three types of service delivery points were included in the survey: public health centers, clinics and hospitals (hereafter public outlets); private health centers, clinics and hospitals (hereafter private outlets); and all types of pharmacies offering more than condoms. This set of outlets was followed one or two times between 2019 and 2020. In Kenya, initial data were collected in April-May 2019, with two rounds of follow-up in September 2019 and in November-December 2019. In Uganda, initial data were collected in October 2019. Follow-up in Uganda was interrupted by the COVID-19 pandemic, so only one round of follow-up data was collected in January–February 2020 (25).

Nested within each outer ring, all households in an inner ring were enumerated, and one-third to one-fourth of all households were randomly selected for survey at each follow-up round. As with the outlet, household data collection was interrupted in Uganda, so three rounds of household data were collected in Kenya, and two rounds were collected in Uganda. At each selected household, one woman was randomly selected for interview from all eligible household members, with sample size targeted to collect data from 200 family planning users per site per round (26). As part of the survey, women who were currently using or who had used contraception in the last 12 months were asked the name of the outlet where they obtained their most recent method. Households were then linked to the outlet they visited. Travel time, least-cost path (accounting for travel barriers, travel speed and actual mode of transport) and Euclidean distances were calculated between the household and the linked outlet. In addition, Euclidean and least-cost path distances were computed from the households to all other outlets within their geographic ring under plausible assumptions (16). The CM4FP study design is described in greater detail elsewhere (24).

In Kenya, 1,282 women aged 18–24 were surveyed, of whom 795 reported use of a modern method within the last 12 months and 272 (34.2%) were linked to the outlet where they obtained their method. Among women aged 25–29, 2,534 were surveyed, of whom 1,638 reported use of a modern method within the last 12 months and 711 (43.4%) were linked to an outlet. In Uganda, 1,004 women aged 18–24 were surveyed, of whom 574 used a modern method within the last 12 months and 184 (32.1%) were linked to an outlet. Among women aged 25–49, 1,987 were surveyed, of whom 1,028 used a modern method within the last 12 months and 418 (40.7%) were linked to an outlet. In both locations, we included all women who matched to an outlet, regardless of whether they reported that they traveled to the outlet from home or from another location, in order to compare each woman's residential supply environment, and to maximize the sample size among younger women.

In Kenya, most surveyed outlets were pharmacies (*n* = 921), followed by private outlets (*n* = 614) and public outlets (*n* = 233). By contrast, the majority of surveyed outlets in Uganda were private outlets (*n* = 826). Only 56 public outlets and 18 pharmacies were surveyed.

### 2.3. Study ethics

Before study participation, enumerators read an informed consent form to all eligible participants in the preferred local language of the participant. Participants were given the opportunity to ask questions, and if they agreed to participate, the enumerator acknowledged consent with a digital signature. Outlets received a small gift of outlet supplies and household survey respondents received an airtime card ranging in value from $2–$2.50 for each completed survey (26).

The Kenya outlet and household surveys were reviewed by the African Medical and Research Foundation Ethics and Scientific Review Committee (AMREF ESRC) and approved on April 8, 2019 (IRB approval code P615-2019). The Uganda surveys were reviewed by the Uganda National Council for Science and Technology (UNCST) and Mildmay Uganda Research and Ethics Committee (MUREC). The Uganda outlet survey was approved on August 30, 2019 (UNSCT approval: SS 5041; MUREC approval: 1005–2019). The Uganda household survey was approved October 25, 2019 (UNCST approval: SS5104; MUREC approval: 1105–2019). The secondary data analysis described here was reviewed by the Institutional Review Board at the University of North Carolina at Chapel Hill (Reference ID 365197), which determined that the study did not constitute human subjects research and was exempt from IRB approval.

### 2.4. Variables

We used the data from the repeated cross-sectional household surveys to consider demographic characteristics (age, education, religion, parity, marital status, and national wealth score) and contraceptive method use (no method, traditional method, short-acting modern method, and long-acting modern method) among female survey respondents in Kenya and Uganda. We defined modern method users to include both current users and users within the last 12 months. Long-acting methods included implants and IUD/copper T. Short-acting methods included injectables, oral contraceptive pills, emergency contraception, standard days/cycle beads, male condoms, female condoms, diaphragm, and foam/jelly. Traditional methods included calendar/rhythm methods, withdrawal, and lactational amenorrhea. Sterilization was excluded from this analysis because there were no sterilizations among respondents aged 18–24. We used outlet data to characterize the supply side environment within a given distance of each respondent. We estimated the mean number of pharmacies, mean number of public outlets, mean number of outlets offering outreach, and mean number of contraceptive methods available for each household related to all outlets within the examined distance, similar to other analyses with outlet and individual-level data (13, 27, 28).

Distances were calculated between surveyed households and each outlet within the outer ring. While both least cost and Euclidean distances were available, we used Euclidean distances for this analysis. Euclidean distance is not an exact proxy for access because it does not address transportation networks or physical barriers between starting and end points, nor does it account for the route used, as it assumes straight line distance. Furthermore, for Euclidean and other spatial access metrics, distance is calculated to the closest outlet rather than the outlet that was used. Despite of these limitations, in this study area, previous analyses focusing on comparisons between spatial access metrics have shown that least cost and Euclidean distance were similar in settings with a high density of road network and outlets (16). As a result, we chose Euclidean distance since it is comparable to least-cost distance and is simple for policymakers to understand. Euclidean distances were computed using the “near” function of the Proximity toolbox in ArcMap (Version 10.5) (29), while all travel time estimates and least cost distances were derived using AccessMod software alpha (Version 5.7.8) (30). Data management was done using STATA (Version 15.0) (31). We included all outlets within a 3 km household radius in Kenya, and all outlets within a 2 km radius in Uganda because of the very high number of private outlets in Uganda.

This analysis is strengthened by the ability to match users to the outlet where they received the method (chosen outlet); while not all women were able to be matched, those who matched provide additional information on outlet characteristics associated with outlet selection. For each country, we compared characteristics of adolescent and youth respondents aged 18–24 to respondents aged 25–49 by method used and outlet matching ability.

We used the outlet audit to compare the characteristics of public outlets, private outlets, and pharmacies in each country. We also used the linked household and outlet data to consider the characteristics of the nearest health outlet offering FP and the chosen (i.e., matched) health outlet for FP among modern method users aged 18–24 and aged 25–49 in each country. We considered several characteristics related to components of structural quality such as the median price for oral contraceptives (in local currency), median price for injection (in local currency), number of methods offered, availability of at least one short acting method, availability of at least one long acting method, stockouts of oral contraceptives at the time of survey, stockouts of injectables at the time of survey, if the outlet conducted FP outreach, if the outlet hosted FP outreach events, whether the outlet was open seven days per week, if the outlet had been providing FP for at least one year, if the outlet supported, coordinated, or supervised community health workers (CHW), provision of FP counseling at the outlet, FP job aids present, if the outlet had a provider who had been trained in the last one year, and clear FP signage present showing services and products.

### 2.5. Estimation strategy

Our estimation strategy has two components. First, we estimate multinomial models for the determinants of contraceptive method choice among the full population—matched users, unmatched users, as well as non-users. In addition to providing substantive information on which factors are important determinants of method choice, this first step also allows us to correct for selection into a mixed logit model for outlet choice which can only be estimated for modern users who can be linked to the outlet where they go to obtain their chosen method. The estimation strategy described below closely mirrors the methodology used by Cronin, Guilkey, and Speizer (13).

The multinomial logit model takes on the following form:

$$\ln\left[\frac{\;p(y_{i}=n|x)}{\;p(y_{i}=1|x)}\right]=x_{i1}\beta_{1n}+x_{i2}\beta_{2n}+\ldots +x_{ik}\beta_{kn}$$

where the dependent variable is the log odds that respondent i (*i* = 1,2…,*N*) makes choice n relative to choice 1. Respondents make one of six choices: no method, traditional method, long-acting method not linked to an outlet, short acting method not linked to an outlet, long-acting method linked to an outlet, and short acting method linked to an outlet. The x's represent individual level characteristics such as age, education, and fertility history. In addition, since each respondent is linked to every outlet in the outer ring surrounding the household sampling frame, we can also include variables such as the average number of methods available at outlets within a certain distance of the respondent's residence. After estimation, we can predict the probability that each respondent is in the two linked modern use categories (long-acting method linked to an outlet and short acting method linked to an outlet) and then calculate the inverse probability weights (IPW) which can be used as weights in the mixed logit model that determines outlet choice. This selection on observables strategy is discussed in more detail in Cronin, Guilkey, and Speizer (13) as well as by Wooldridge (31, 32) and Imbens (33).

The data sets for this analysis are rich because location identifiers were collected for all outlets and all eligible households, permitting calculation of the Euclidean distance to every outlet from each household. This allows us to consider characteristics of the outlets in each respondent's supply environment, such as whether they have trained providers, the number of methods available and prices for different contraceptive methods. This also allows us to consider distances of individual households to outlets, which is preferable to a common data collection strategy of simply collecting data on the closest outlet of each type to a cluster or household. In fact, Cronin, Guilkey, and Speizer (13) show that just having information on the closest outlets leads to severely biased results.

Our estimation strategy for the outlet choice equations was as follows. Using IPW, we first used the standard conditional logit estimator as a preliminary step to screen the large number of outlet characteristics since, as expected, many were highly collinear. This strategy of first overfitting the model and then dropping variables seemed reasonable since omitting a relevant variable is a much more severe specification error than including an irrelevant variable. Dropping variables with little explanatory power was important because of the fairly small number of individuals that could be linked to outlets. With the reduced set of variables we then estimated models using both conditional logit and mixed logit and then used likelihood ratio tests to see if the mixed logit results were significantly better than conditional logit. For all four samples (by country and age group), the null of no difference was rejected with a *p*-value close to zero and so we only present the mixed logit results.

The specific estimation we use is the mixed logit model which is a random coefficient conditional logit model that assumes that utility can be written as a function of outlet characteristics:

$$U_{im}=z_{im1}\beta_{1i}+z_{im2}\beta_{2i}+\ldots +z_{im\ell }\beta_{\ell i}+\varepsilon_{im}.$$

where the dependent variable is the utility that individual i receives from outlet m with *m* = 1,2,…,*M_c_* where *M_c_* is the total number of outlets within 3 km in Kenya and within 2 km in Uganda *c* = 1,2,3,4 (there are four counties or districts in the data set for each country). The z's represent outlet specific attributes and the $\varepsilon^{\prime }s$ represent a random error where we make the standard assumption that they follow a Type 1 negative extreme value distribution. The $\beta^{\prime }s$ indicate the weight that individual i puts on each attribute in making her choice. We assume that the ℓ×1 vector of $\beta_{i}^{\prime }s$ is distributed as $\beta_{i}∼N(\beta ,\Sigma_{\beta })$ where $\Sigma_{\beta }$ is a ℓ×ℓ covariance matrix. Because of the relatively small sample size for the younger sample, we restrict $\Sigma_{\beta }$ to be a diagonal matrix which leads to more stable parameter estimates but still relaxes the independence of irrelevant alternatives (IIA) assumption for the standard conditional logit model.

Our statistical model for facility choice is based on the conditional logit model where each individual can consider the complete set of facilities in her county for her choice of where to go for contraceptives. This model is attractive because the number of parameters that must be estimated does not increase with the number of choices and the interpretation of the estimated coefficients is the weight that an individual gives to each facility attribute (for example, stock outs and distance to travel) in making her choice. As is true with all categorical outcome models, the estimated coefficients are scale dependent however, we can transform the estimated coefficients into a common metric which is the distance they are willing to travel for each attribute. For example, if a person is willing to travel an additional. 1 of a kilometer to go to a facility with one more method available but they are willing to travel. 2 of a kilometer to go to a facility open seven days a week, then they value the convenience of seven day a week access relative to the convenience of adding one more method to the selection of methods.

## 3. Results

### 3.1. Descriptive results

Table 1 presents the demographic characteristics of surveyed women in Kenya and Uganda. As expected, in both countries, women aged 25–49 were more likely than younger women to be married, to be in the top quintile of the national wealth score, and to be higher parity. In both countries and in both age groups, modern method users were more likely to be married, to be in the top wealth quintile, and to be higher parity. Younger women were more likely than women aged 25–49 to have secondary education or college education. Demographic differences between users and non-users are also similar in Kenya and Uganda. Younger women were more likely to use short-acting contraceptive methods (44.0% vs. 38.9% in Kenya, *p* < 0.01; 42.4% vs. 38.3% in Uganda, *p* = 0.06) and were less likely to use long-acting methods (18.0% vs. 25.7% in Kenya, *p* < 0.01; 12.8% vs. 15.2% in Uganda, *p* = 0.06). Overall method use was slightly lower in Uganda than in Kenya by age group and method type. Access to outlets (bottom of Table 1) was generally similar by age group.

**Table 1:** Characteristics of women in CM4FP household survey in Kenya and Uganda, 2019–2020.

	Kenya						Uganda					
	Women aged 18–24			Women aged 25–49			Women aged 18–24			Women aged 25–49		
		Modern method users in the last year (*n* = 795)			Modern method users in the last year (*n* = 1,638)			Modern method users in the last year (*n* = 530)			Modern method users in the last year (*n* = 1,026)	
	Full sample (*n* = 1,282)	Users not matched to an outlet (*n* = 523)	Users matched to an outlet (*n* = 272)	Full sample (*n* = 2,534)	Users not matched to an outlet (*n* = 927)	Users matched to an outlet (*n* = 711)	Full sample (*n* = 1,004)	Users not matched to an outlet (*n* = 346)	Users matched to an outlet (*n* = 184)	Full sample (*n* = 1,987)	Users not matched to an outlet (*n* = 608)	Users matched to an outlet (*n* = 418)
**Age (%)**												
18–19	16**.**0	12**.**4	9**.**9	N/A	N/A	N/A	19**.**8	16**.**2	11**.**4	N/A	N/A	N/A
20–24	84**.**0	87**.**6	90**.**1	N/A	N/A	N/A	80**.**2	83**.**8	88**.**6	N/A	N/A	N/A
25–29	N/A	N/A	N/A	36**.**7	39**.**3	42**.**9	N/A	N/A	N/A	42**.**5	46**.**6	52**.**6
30–34	N/A	N/A	N/A	25**.**8	27**.**3	27**.**4	N/A	N/A	N/A	25**.**0	27**.**6	24**.**9
35–39	N/A	N/A	N/A	18**.**6	17**.**7	17**.**9	N/A	N/A	N/A	16**.**3	15**.**0	14**.**4
40–44	N/A	N/A	N/A	11**.**5	11**.**4	8**.**3	N/A	N/A	N/A	9**.**4	7**.**2	6**.**0
45–49	N/A	N/A	N/A	7**.**4	4**.**3	3**.**5	N/A	N/A	N/A	6**.**9	3**.**6	2**.**2
**Education (%)**												
None	12**.**2	0**.**4	0**.**4	16**.**3	3**.**9	3**.**8	0**.**5	0**.**3	0**.**0	5**.**1	2**.**1	3**.**4
Primary	21**.**5	22**.**4	34**.**6	33**.**6	35**.**8	42**.**1	32**.**5	30**.**9	33**.**7	35**.**1	31**.**7	35**.**4
Secondary	28**.**3	32**.**5	35**.**7	25**.**6	29**.**8	29**.**0	47**.**2	48**.**3	45**.**7	32**.**4	33**.**6	39**.**7
College	38**.**0	44**.**7	29**.**4**	24**.**5	30**.**5	25**.**2*	19**.**7	20**.**2	20**.**7	27**.**2	32**.**4	21**.**5**
Muslim (%)	6**.**7	3**.**6	6**.**3	8**.**5	7**.**6	9**.**7	15**.**6	13**.**6	17**.**4	15**.**8	13**.**2	20**.**6**
**Parity (%)**												
Parity 0	57**.**2	53**.**4	27**.**9	19**.**9	8**.**3	4**.**4	47**.**1	42**.**5	27**.**2	10**.**0	10**.**9	6**.**2
Parity 1	27**.**9	30**.**4	43**.**4	17**.**2	21**.**6	18**.**4	33**.**2	37**.**3	47**.**8	17**.**1	18**.**6	18**.**9
Parity 2	11**.**1	12**.**6	20**.**6	22**.**5	25**.**1	29**.**7	13**.**8	15**.**3	18**.**5	21**.**4	23**.**7	25**.**1
Parity 3+	3**.**8	3**.**6	8**.**1**	40**.**4	45**.**0	47**.**5**	5**.**9	4**.**9	6**.**5**	51**.**4	46**.**9	49**.**8
Married/in union (%)	42**.**0	46**.**1	65**.**8**	66**.**0	74**.**3	81**.**7**	55**.**9	54**.**6	66**.**3**	71**.**3	74**.**0	71**.**1
Top quintile—equity tool national quintile (%)	39**.**2	42**.**4	41**.**9	51**.**9	61**.**6	57**.**5	62**.**6	65**.**9	70**.**1	69**.**0	76**.**3	68**.**7**
**Method use (%)**												
No method	30**.**2	N/A	N/A	27**.**7	N/A	N/A	29**.**3	N/A	N/A	30**.**5	N/A	N/A
Traditional	7**.**8	N/A	N/A	7**.**6	N/A	N/A	13**.**5	N/A	N/A	14**.**4	N/A	N/A
Short-acting^a^	44**.**0	69**.**6	73**.**5%	38**.**9	46**.**9	77**.**5	40**.**5	77**.**2	76**.**1	36**.**9	64**.**0	82**.**5
Long-acting	18**.**0	30**.**4	26**.**5%	25**.**7	53**.**1	22**.**5**	12**.**3	22**.**8	23**.**9	14**.**7	36**.**0	17**.**5**
Mean # of pharmacies within radius^b^	52**.**20	50**.**33	45**.**26	44**.**84	47**.**19	41**.**83**	10**.**35	10**.**75	10**.**63	10**.**93	11**.**70	11**.**14
Mean # of public facilities within radius	4**.**30	4**.**11	3**.**92	3**.**99	4**.**27	3**.**86*	1**.**70	1**.**76	1**.**73	1**.**80	1**.**95	1**.**86
Mean # of private facilities within radius	N/A	N/A	N/A	N/A	N/A	N/A	64**.**20	65**.**33	70**.**17	63**.**98	67**.**27	68**.**29
Mean # of facilities with outreach within radius	6**.**34	6**.**06	5**.**81	5**.**63	5**.**75	5**.**45	5**.**76	5**.**95	5**.**97	6**.**03	6**.**36	6**.**14
Mean # of methods at facilities within radius	3**.**87	3**.**86	3**.**95**	3**.**85	3**.**82	3**.**90**	3**.**47	3**.**53	3**.**59	3**.**47	3**.**61	3**.**61

N/A, not applicable.

a Short acting methods includes standard days/beads users who were linked to an outlet.

b We included all facilities within a 3 km household radius in Kenya, and all facilities within a 2 km radius in Uganda.

* *p* < 0.05 in comparisons between women matched to an outlet and women not matched to an outlet.

** *p* < 0.01 in comparisons between women matched to an outlet and women not matched to an outlet.

Table 1 also compares users of any modern method who are matched and not matched to an outlet. Among women aged 18–24 and aged 25–49 in Kenya and among women aged 25–49 in Uganda, users who were not matched to an outlet were more likely to be college educated. Among both age groups in Kenya and among younger women in Uganda, unmatched users were less likely to be married and more likely to be lower parity. Among women aged 25–49 in both countries, unmatched women were more likely to be users of long-acting methods, which may reflect the passage of time since method placement and possibly women's mobility. The mean number of outlets accessible were generally the same between unmatched and matched users in both countries. In Kenya only, on average, there were more methods available at outlets within 3 km of Euclidean distance of users with matched outlets than for users in unmatched outlets.

Table 2 compares the characteristics of public outlets, private outlets, and pharmacies in the outlet audit in Kenya and Uganda. In both countries, public outlets were the least expensive source of both oral contraceptives and injections. Pharmacies were the most expensive source of oral contraceptives, and private outlets were the most expensive source for injections. Public outlets offered the largest number of methods (4.70 methods in public outlets compared to 4.35 in private outlets and 3.23 in pharmacies in Kenya; and 4.30 methods in public outlets compared to 3.57 in private outlets and 3.35 in pharmacies in Uganda). Nearly all audited outlets in both countries offered at least one short acting method. Long-acting methods were only available in about 52.9% of public outlets and 63.2% of private outlets in Kenya and in 44.6% of public outlets and 24.8% of private outlets in Uganda. Not surprisingly, long-acting methods were rarely available in pharmacies in either country. In both countries, pharmacies and private outlets were more likely to be open seven days per week (51.6% of pharmacies and 51.0% of private facilities compared to 13.0% of public outlets in Kenya; and 86.4% of pharmacies and 81.1% of private outlets compared to 60.7% of public outlets in Uganda). On the other hand, public outlets were much more likely to conduct FP outreach; support, coordinate, or supervise CHW; offer FP counseling; have FP job aids; have a provider trained in the last one year; and have visible FP signage.

**Table 2:** Characteristics of all outlets in CM4FP audit in Kenya and Uganda, 2019–2020.

	Kenya			Uganda		
	Public (*n* = 233)	Private (*n* = 614)	Pharmacy (*n* = 921)	Public (*n* = 56)	Private (*n* = 826)	Pharmacy (*n* = 18)
Median price for oral contraceptives (local currency)	2.02	40.41	102.70	160.71	1,038.84	2,128.32
Median price for injection (local currency)	3.15	113.03	18.72	285.71	2,333.84	466.10
Number of methods offered	4.70	4.35	3.23	4.30	3.57	3.35
At least 1 short acting method available (%)	99.6	96.3	99.7	100.0	97.8	100.0
At least 1 LARC available (%)	52.9	63.2	1.3	44.6	24.8	4.2
Stock out of oral contraceptives at time of survey (%)	10.8	12.1	12.5	25.0	24.9	21.2
Stock out of injectables at time of survey (%)	5.4	19.4	20.2	23.2	20.5	12.7
Conducts FP outreach (%)	64.6	14.0	0.7	75.0	9.0	1.7
Hosts FP outreach events (%)	24.2	15.5	0.7	32.1	6.1	1.7
Open 7 days/week (%)	13.0	51.0	51.6	60.7	81.1	86.4
Outlet provided FP for ≥1 year (%)	83.9	77.0	79.9	85.7	69.9	69.5
Outlet supports, coordinates, or supervises CHW (%)	28.3	10.7	0.3	17.9	4.4	0.0
Outlet counsels clients on FP (%)	97.3	91.2	75.8	96.4	79.2	52.5
Outlet has FP job aids (%)	65.9	41.5	12.8	78.6	19.6	5.1
Outlet has a provider that has been trained on FP in the last year (%)	45.3	38.8	23.9	53.6	31.0	35.6
Clear FP signage present showing services and products (%)	35.9	36.3	17.0	21.4	12.6	0.0

Stockouts were more common in Uganda, ranging from 12.7% to 25.0%, compared to 5.4% to 20.2% in Kenya. In Kenya, stockouts of injectables at the time of survey were less common in public outlets (5.4%) than in private outlets (19.4%) and pharmacies (20.2%). In Uganda, stockouts of injectables at the time of survey were less common in pharmacies (12.7%) than in public outlets (23.2%) or private outlets (20.5%).

Table 3 compares the characteristics of the nearest outlet offering FP to the characteristics of the chosen outlet for FP by age group in each country. In both countries, the mean Euclidean distance traveled was greater for the chosen outlet than for the nearest outlet (1.76 km vs. 0.48 km among women aged 18–24 in Kenya; 1.71 km vs. 0.55 km among women aged 25–49 in Kenya; 1.29 km vs. 0.29 km among women aged 18–24 in Uganda; and 1.28 km vs. 0.28 km among women aged 25–49 in Uganda). In Kenya, public outlets were more likely to be the chosen outlet than the nearest outlet in both age groups, while pharmacies were more likely to be the nearest outlet than the chosen outlet. In Uganda, pharmacies and public outlets were more likely to be the chosen outlet than the nearest outlet, while private outlets were more likely to be the nearest outlet than the chosen outlet. This may be explained in part by unavailability of chosen methods at the nearest outlet (i.e., women selecting a long-acting method may need to bypass a nearby pharmacy or private outlet if it does not offer that method.)

**Table 3:** Characteristics of nearest and chosen facilities in CM4FP audit in Kenya and Uganda.

	Kenya				Uganda			
	Women aged 18–24 (*n* = 272)		Women aged 25–49 (*n* = 711)		Women aged 18–24 (*n* = 184)		Women aged 25–49 (*n* = 418)	
	Nearest FP outlet	Chosen FP outlet	Nearest FP outlet	Chosen FP outlet	Nearest FP outlet	Chosen FP outlet	Nearest FP outlet	Chosen FP outlet
**Type of outlet (%)**								
Private	N/A	N/A	N/A	N/A	92.9	37.0	90.4	43.8
Public	25.4	56.3	27.4	56.7	2.7	46.7	2.4	41.1
Pharmacy	51.1	21.7%	46.6	15.9	4.3	16.3	7.2	15.1
Average distance (km)	0.48	1.76	0.55	1.71	0.29	1.29	0.28	1.28
Median price for oral contraceptives (local currency)	59.94	37.01	47.45	31.46	1,141.30	834.24	1,327.75	958.13
Median price for injection (local currency)	30.00	27.44	30.11	31.89	2,720.11	1,288.04	2,550.24	1,261.96
Number of methods offered	4.51	5.84	4.45	5.91	3.74	5.10	3.74	5.11
At least 1 short acting method available (%)	100.0	99.6	99.9	100.0	98.4	99.5	98.3	99.8
At least 1 LARC available (%)	48.5	76.5	49.2	79.9	21.2	67.4	19.1	69.4
Stock out of oral contraceptives at the time of survey (%)	4.0	4.8	8.0	3.2	31.5	8.7	27.0	12.4
Stock out of injectables at the time of survey	11.4	8.8	14.5	10.3	20.1	5.4	17.2	9.6
Conducts FP outreach (%)	19.9	48.9	23.6	48.7	7.6	46.2	5.5	40.9
Hosts FP outreach events (%)	12.9	47.4	12.2	49.2	9.8	36.4	9.6	33.7
Open 7 days/week (%)	40.1	28.7	36.7	25.0	95.7	74.5	95.%	70.6
Outlet provided FP for ≥1 year (%)	75.0	89.0	70.6	89.2	59.2	51.1	66.5	56.9
Outlet supports, coordinates, or supervises CHW (%)	24.3	31.3	26.3%	35.6%	10.9%	22.8%	11.7%	22.2%
Outlet counsels clients on (%)	83.1	94.1	82.4%	95.2%	76.6%	75.5%	73.7%	77.3%
Outlet has FP job aids (%)	34.6	70.6	38.1%	73.1%	23.9%	46.7%	26.1%	50.0%
Outlet has a provider that has been trained on FP in the last year (%)	28.7	57.0%	31.5%	56.5%	21.7%	37.5%	23.9%	34.0%
Clear FP signage present showing services and products (%)	23.5	62.1%	26.6%	61.7%	9.2%	22.8%	9.1%	23.0%

In both countries, compared to the nearest outlet, the chosen outlet was more likely to have lower prices for oral contraceptives, fewer stockouts of injectables at the time of survey, conduct FP outreach, host FP outreach events, support, coordinate, or supervise CHWs, provide FP job aids, have a provider who has been trained in the last one year, and display FP signage. In both countries, the chosen outlet was less likely to be open seven days per week. Prices for injection were lower at chosen health outlets for both age groups in Uganda, but only for younger users in Kenya. Stockouts of oral contraceptives were less common at chosen outlets in both age groups in Uganda, but only for users aged 25–49 in Kenya.

### 3.2. Multinomial and mixed logit results

Multinomial logit estimates were used to create inverse probability weights that were fed into the outlet choice models. Supplementary Materials S1, S2 that include Supplementary Tables S1, S2 present the multinomial results and discussion for Kenya for both age groups (18–24 and 25–49) and Uganda, respectively. Overall, with the exception of weaker results for the younger age group for Uganda, the results for both age groups and for both countries are reasonable for use in the construction of inverse probability weights (IPW).

We present the results of mixed logit models with IPW to correct for selection in Supplementary Tables S3, S4. Supplementary Table S3 presents the results predicting outlet choice for Kenya for both age groups. As with multinomial logit, the estimated coefficients can only be interpreted in terms of sign and significance. Therefore, we mainly focus on the willingness to travel results which are not scale dependent (see Tables 4a, 4b). Hence, we can make comparisons across age groups and countries. For Kenya, based on the mixed logit models presented in Supplementary Table S3, for the younger sample, we see that three of the estimated coefficients had significant standard deviations (the “SD” results in the lower part of the table), meaning that the effect of these variables on outlet choice was variable across the set of respondents. For example, while the coefficient for distance is negative and highly significant, the large standard deviation indicates that some individuals did not have much of an aversion to travel—presumably to go to a higher quality outlet. However, there were also individuals where distance was a prohibitive barrier. The other two coefficients that were random were the number of methods offered and the indicator that the outlet was public. We estimated the same model for respondents aged 25–49 and again found that the same subset of the variables had random coefficients that were highly significant. In terms of main effects, open seven days a week and the pharmacy indicator were not significant at any standard level.

**Table 4a:** Willingness to travel (in km) for outlet characteristics among women aged 18–24 and aged 25–49, CM4FP household survey in Kenya.

	Women aged 18–24 (*n* = 272)		Women aged 25–49 (*n* = 711)	
	Willingness to travel (km)^a^	95%CI	Willingness to travel (km)^a^	95% CI
Methods offered	0.12	0.00**,** 0.23	0.16	0.09, 0.23
Open 7 days/week	0.27	−0.06, 0.59	0.13	−0.04, 0.29
Stockout of injectables at the time of survey	−0.15	−0.42, 0.12	−0.01	−0.16, 0.14
Outlet has a provider that has been trained on FP in the last year	0.17	−0.07, 0.42	0.40	0.24, 0.57
Clear FP signage present showing services and products	0.41	0.17, 0.65	0.61	0.34, 0.87
Public	2.17	0.03, 4.32	2.14	1.47, 2.82
Pharmacy	0.48	0.16, 0.80	−6.04	−12.67, 0.58

^a^ Willingness to travel = willingness to travel (in Euclidean km) for a given facility characteristic.

**Table 4b:** Willingness to travel (in km) for outlet characteristics among women aged 18–24 and aged 25–49, CM4FP household survey in Uganda.

	Women aged 18–24 (*n* = 184)		Women aged 25–49 (*n* = 418)	
	Willingness to travel (km)^a^	95%CI	Willingness to travel (km)^a^	95% CI
Methods offered	0.29	0.09**,** 0.49	0.32	0.22, 0.42
Open 7 days/week	0.58	−0.49, 1.64	0.20	−0.49, 0.89
Stockout of injectables at the time of survey	−0.53	−1.13, 0.07	−0.29	−0.56, −0.03
Outlet has a provider that has been trained on FP in the last year	0.26	−0.05, 0.56	−0.15	−0.34, 0.04
Clear FP signage present showing services and products	0.20	−0.11, 0.51	0.33	0.14, 0.51
Public	2.00	1.08, 2.91	1.45	1.09, 1.81
Pharmacy	1.26	0.56, 1.97	0.98	0.70, 1.26

^a^ Willingness to travel = willingness to travel (in Euclidean km) for a given facility characteristic.

The results of mixed logit models with selection correction for Uganda are in Supplementary Table S4. For the younger respondents, the results are not as strong as they were for Kenya. However, we again see strong distance effects but it is the only variable that has a strongly significant “SD” whereas the “SD” for open seven days a week is significant but only at the 10% level. We do see that stock outs of injectables have a negative effect and a *p*-value of.057. This variable was insignificant for Kenya and was dropped from the final model. As expected, the results for the respondents aged 25–49 are much stronger and injection stock outs is now significant at the 5% level. An interesting result is that the coefficient open seven days a week is not significant at any standard level, however, the estimated standard deviation is highly significant, indicating that this variable is important for a subset of the respondents.

We next present the willingness to travel results calculated from the mixed logit models among users who matched to an outlet. As described above, calculated willingness to travel accounts for selection into one of the matched categories by using IPW. Tables 4a, 4b present the willingness to travel, in Euclidean km, for outlet characteristics, in Kenya and Uganda respectively. These results show similarities across countries and age groups as well as some distinctions by age group within countries. For example, in both countries and in both age groups, users were willing to travel further to outlets offering more methods (0.12 km among users aged 18–24 in Kenya; 0.16 km for users aged 25–49 in Kenya; 0.29 km for users aged 18–24 in Uganda; and 0.32 km for users aged 25–49 in Uganda for each additional method.) Women in both age groups in Kenya and only women aged 25–49 in Uganda were willing to travel further to outlets with visible FP signage (0.41 km for users aged 18–24 in Kenya; 0.61 km for users aged 25–49 in Kenya; and 0.33 km for users aged 25–49 in Uganda.) Women in both age groups in both countries were willing to travel further for public outlets (Kenya: 2.17 km among users aged 18–24 and 2.14 km among users aged 25–49; Uganda: 2.00 km among users aged 18–24 and 1.45 km among users aged 25–49). Only younger women in Kenya and women in both age groups in Uganda were willing to travel further to a pharmacy (0.48 km among users aged 18–24 in Kenya; 1.26 km among users aged 18–24 in Uganda; and 0.98 km among users aged 25–49 in Uganda). In Uganda among women aged 25–49 only, women were less likely to travel to an outlet that experienced stockouts of injectables (−0.29 km). In Kenya among women aged 25–49 only, women were willing to travel 0.40 km to visit an outlet with a provider who was trained in the last one year.

## 4. Discussion

Our results suggest that women are willing to travel further and bypass closer outlets for outlets with higher quality and characteristics that they value. These findings are similar to other studies where women choose specific outlets for quality characteristics (12–15, 27). Some characteristics, such as cost and method availability, were important across countries and across age groups. Surveyed women in both countries and both age groups were willing to travel further for public sector outlets. In a recent analysis of nationally representative data in Kenya, over half of public sector users reported receiving free FP, while less than 11% of private facility users and less than 1% of pharmacy users received free FP (35). This suggests that lower cost or free services may be important in outlet selection. Women were also willing to travel further to outlets offering more methods, irrespective of age. In addition, in Uganda only among women aged 25–49, women were less willing to travel to outlets experiencing stockouts of injectables. This points to the importance of reliable availability of highly used methods such as the injectable.

Other outlet characteristics were context or age specific. For example, most women in both countries were willing to travel further to a pharmacy than to a private outlet, but this result was not significant for women aged 25–49 in Kenya. The greater willingness to travel to a pharmacy may suggest the desire for privacy in the face of stigma surrounding FP. This would be consistent with qualitative research finding that unmarried women preferred to obtain their family planning methods from pharmacies or chemists due to privacy concerns (36).

Strengths of this study are our ability to examine the characteristics of the outlets that women actually visited to obtain their contraceptive method, rather than assuming that women visit the closest outlet or creating a supply environment with the characteristics of a small number of nearby outlets. We are also able to describe the characteristics of the full set of outlets within an FP user's supply environment and then compare those characteristics to the characteristics of the chosen outlet. Our distance variables represent Euclidean distances between women's residences and each outlet, instead of assigning the distance between a cluster centroid and an outlet to every respondent in a sampled cluster. The use of measured distances between individual residences and outlets allows for greater measurement precision than estimating distances from the center of a sampled cluster to an outlet. This is particularly important in studies conducted in urban areas where there might be multiple outlets within a relatively short distance of a sampled cluster. In these cases, use of distance between a cluster centroid and an outlet could lead to incorrect assignment of the closest outlet to a woman's residence.

There are limitations to this study as well. First, the survey questions did not include some of the key quality features, such as provider bias, that matter, particularly for younger women. Future studies would benefit from qualitative data collection to better understand women's reported priorities for qualities of services. Men's data were not available for this analysis. While we acknowledge the important role of men in contraceptive use, we examined the role of stockouts in facility choice. Further, the facility-based methods that we focus on in this analysis including oral contraceptive pills, injections, and implants, are adopted by women and thus women's responses will be more accurate to reflect the service delivery environment around stockouts, signage and interactions with providers. In addition, implants and oral contraceptives are much more likely to be subject to stockouts than condoms in our study area (37).

The distance calculations also assumed that women took the most direct, Euclidean, route to an outlet, and that they were traveling from home, rather than from their workplace or another convenient location. Spatial accessibility can be proxied by different measures such as Euclidean distance, cost distance and reported travel time. Euclidean distance may not always represent the amount of time required to travel between their residence and a contraceptive outlet. It neither captures the route used, as it assumes a straight-line path, nor does it account for any physical barriers between the two locations. We therefore considered both time cost and Euclidean distance (not shown). In time cost only models, time cost was significant in the expected direction. However, in models including both time cost and distance, distance was strongly significant and time cost was insignificant. We conjectured that, while time cost may be intuitively appealing, it is highly correlated with distance. Indeed, specific to this study area, Bouanchaud et al. have shown that Euclidean distance gives similar results to modelled least cost distances (accounting for travel barriers and actual mode of transport used) in these small urban to semiurban settings with good road networks and high FP outlet density (34). In addition, Euclidean distance is less subject to measurement error. There was also evidence of heaping around certain times (10, 20, 30 min) for the reported travel time measure. Further, using Euclidean distance allowed us to calculate distance between a participants' residence and every contraceptive outlet in their outer ring. Therefore, we employ Euclidean distances as they were comparable to distance that accounted for barriers and more consistently measurable.

Finally, the survey was not designed to be population representative, so results cannot necessarily be generalized to the wider population, or the region more broadly. While we tried to address selection into nonuse and missingness using IPW, this was limited because a number of users did not match to an outlet. This led to a small number of respondents in our willingness to travel analyses, especially among young users in Uganda.

In conclusion, this study demonstrated the importance of outlet quality and desirable characteristics in outlet selection. This has important implications for FP uptake and use. For example, our results suggest the relative desirability of pharmacies compared to private outlets, especially among younger women. This may be in part due to stigma and a need for privacy in this population but may result in use of less effective methods due to their wider availability at pharmacies. Programs seeking to expand access to FP should therefore be encouraged to ensure access to outlets providing high quality services, including highly visible signage, consistently available methods, and bias-free provision of services.

## Data Availability

The original contributions presented in the study are included in the article/**Supplementary Materials**, further inquiries can be directed to the corresponding author. The data are available at https://go.pardot.com/l/857593/2021-09-15/3nqcv.
