# Supplementary material for: Choice of a family planning outlet in urban areas: The role of distance and quality of services in Kenya and Uganda
Source: Front Glob Womens Health. 2023 Mar 30;4:1117849. doi: 10.3389/fgwh.2023.1117849 (PMC10099502; doi:10.3389/fgwh.2023.1117849)
Supplement: Supplementary file 1 [file Table1.docx]

**Supplemental Materials 1: Results of multnomial logistic regression predicting method use among women aged 18-24 and aged 25-49 in the CM4FP household survey in Kenya, 2019-2020**

In this section, we present the results of the multinomial logit models estimating determinants of contraceptive method choice among matched users, unmatched users, and non-users in Kenya. We used non-use as the base category, so the coefficients can be interpreted as the effect of each explanatory variable on the log odds of being in one of the other method choice categories relative to being a non-user. We discuss the results for the younger respondents first. The reference category for age is 18 or 19 years old and we see that being in the older age category (20 to 24) has a consistently positive and significant effect of being in any of the other choice categories at least at the 5% level except for traditional method use where the p-value is slightly higher than 0.05 and for the linked individuals using long-acting methods – the smallest category – where age is not significant at any level. The no education dummy is sporadically significant and when it is, it is in the expected direction making it less likely that a respondent with no education is using a method relative to being a non-user. The same is true for the Muslim dummy which reduces the log odds of using a method relative to non-use when it is significant. The omitted category for parity is no children and we see that being in one of the other parity categories typically increases the likelihood of using a method relative to non-use when the coefficients are significant which is most of the time. Note that the fertility categories are likely endogenous to contraceptive method choice, however, this is not a problem and is actually preferred when used to construct inverse probability weights (see, for example, Wooldridge, 2007). Marital status is also potentially endogenous and we see mostly positive and significant effects for being currently married on the log odds of using a method. Finally, in a few cases, the average number of methods available in outlets within three kilometers has a positive and significant effect on long-acting method use and short-acting method use among women who linked to an outlet.

The results for the older age group as a whole are stronger than for the younger age group as would be expected with the larger sample size with more users. The omitted category for age is 25-29 and we see that being in one of the older age categories typically has a negative effect on being in one of the use categories relative to non-use. Most of the other effects are consistent with the results for the younger age group. Being in the highest wealth quintile is significant or marginally and positive in a few cases and we see that the availability of outlets, outreach and methods are more often significant and have stronger positive effects for the older age group relative to the younger one.

**Table S1: Multinomial logistic regression predicting method use among women aged 18-24 and aged 25-49 in the CM4FP household survey in Kenya, 2019-2020**

|  |  | **Age 18-24** |  | | **Age 25-49** |  | |
| --- | --- | --- | --- | --- | --- | --- | --- |
|  |  | **Coefficient** | **95%CI** | | **Coefficient** | **95% CI** | |
| None (base outcome | |  | |  |  |  | |
| Traditional | |  |  |  |  |  |  |
|  | Age 20-24 (ref 18-19) | 0.64 | -0.02 | 1.30 | N/A | N/A | N/A |
|  | 30-34 (ref 25-29) | N/A | N/A | N/A | -0.01 | -0.50 | 0.49 |
|  | 35-39 (ref 25-29) | N/A | N/A | N/A | -0.32 | -0.86 | 0.21 |
|  | 40-44 (ref 25-29) | N/A | N/A | N/A | -0.69 | -1.34 | -0.04 |
|  | 45-49 (ref 25-29 | N/A | N/A | N/A | -0.94 | -1.63 | -0.25 |
|  | No education | -17.69 | -1910.28 | 1874.89 | -2.25 | -3.17 | -1.34 |
|  | Muslim religion | -0.35 | -1.13 | 0.43 | -0.15 | -0.73 | 0.42 |
|  | Parity 1 | 0.64 | 0.03 | 1.25 | 0.34 | -0.36 | 1.03 |
|  | Parity 2 | 0.62 | -0.41 | 1.66 | 0.75 | 0.07 | 1.43 |
|  | Parity 2 or more | 0.29 | -1.29 | 1.88 | 1.29 | 0.61 | 1.96 |
|  | Married/in union | 0.77 | 0.19 | 1.35 | 0.95 | 0.50 | 1.41 |
|  | Top quintile -- national wealth score | -0.29 | -0.81 | 0.22 | 0.47 | 0.04 | 0.91 |
|  | Number of pharmacies within 3 km | 0.02 | -0.01 | 0.05 | -0.02 | -0.05 | 0.00 |
|  | Number of public facilities within 3 km | -0.17 | -0.35 | 0.00 | 0.09 | -0.03 | 0.22 |
|  | Number of outreach facilities within 3 km | -0.07 | -0.30 | 0.16 | 0.20 | 0.02 | 0.38 |
|  | Mean number of methods at facilities within 3 km | 0.17 | -0.60 | 0.95 | 0.01 | -0.45 | 0.47 |
|  | Constant | -2.38 | -5.29 | 0.53 | -2.60 | -4.36 | -0.84 |
| Long acting methods, not linked to a facility | | |  |  |  |  |  |
|  | Age 20-24 (ref 18-19) | 0.80 | 0.09 | 1.52 | N/A | N/A | N/A |
|  | 30-34 (ref 25-29) | N/A | N/A | N/A | -0.51 | -0.89 | -0.12 |
|  | 35-39 (ref 25-29) | N/A | N/A | N/A | -1.08 | -1.51 | -0.66 |
|  | 40-44 (ref 25-29) | N/A | N/A | N/A | -1.08 | -1.56 | -0.60 |
|  | 45-49 (ref 25-29 | N/A | N/A | N/A | -2.23 | -2.84 | -1.62 |
|  | No education | -2.44 | -4.07 | -0.80 | -1.10 | -1.65 | -0.55 |
|  | Muslim religion | -1.78 | -2.76 | -0.80 | -0.70 | -1.19 | -0.21 |
|  | Parity 1 | 2.99 | 2.23 | 3.75 | 2.64 | 1.82 | 3.46 |
|  | Parity 2 | 3.48 | 2.49 | 4.47 | 3.36 | 2.55 | 4.16 |
|  | Parity 2 or more | 2.96 | 1.66 | 4.26 | 3.81 | 3.00 | 4.62 |
|  | Married/in union | 0.76 | 0.20 | 1.33 | 0.80 | 0.46 | 1.14 |
|  | Top quintile -- national wealth score | -0.33 | -0.85 | 0.19 | 0.44 | 0.10 | 0.79 |
|  | Number of pharmacies within 3 km | 0.02 | -0.01 | 0.05 | -0.02 | -0.04 | 0.00 |
|  | Number of public facilities within 3 km | -0.20 | -0.38 | -0.02 | 0.05 | -0.05 | 0.16 |
|  | Number of outreach facilities within 3 km | -0.17 | -0.40 | 0.06 | 0.21 | 0.07 | 0.35 |
|  | Mean number of methods at facilities within 3 km | 0.74 | 0.10 | 1.38 | -0.13 | -0.48 | 0.21 |
|  | Constant | -5.35 | -7.82 | -2.88 | -2.89 | -4.32 | -1.46 |
| Short acting methods, not linked to a facility | | |  |  |  |  |  |
|  | Age 20-24 (ref 18-19) | 0.83 | 0.40 | 1.27 | N/A | N/A | N/A |
|  | 30-34 (ref 25-29) | N/A | N/A | N/A | -0.25 | -0.63 | 0.13 |
|  | 35-39 (ref 25-29) | N/A | N/A | N/A | -0.78 | -1.21 | -0.35 |
|  | 40-44 (ref 25-29) | N/A | N/A | N/A | -0.83 | -1.32 | -0.34 |
|  | 45-49 (ref 25-29 | N/A | N/A | N/A | -1.76 | -2.36 | -1.15 |
|  | No education | -18.16 | -1019.28 | 982.96 | -2.85 | -3.55 | -2.16 |
|  | Muslim religion | -1.77 | -2.49 | -1.06 | -0.56 | -1.10 | -0.03 |
|  | Parity 1 | -0.10 | -0.56 | 0.37 | 0.67 | 0.20 | 1.13 |
|  | Parity 2 | 0.04 | -0.80 | 0.87 | 0.61 | 0.12 | 1.09 |
|  | Parity 2 or more | -0.88 | -2.30 | 0.55 | 0.97 | 0.47 | 1.47 |
|  | Married/in union | 0.29 | -0.14 | 0.71 | 0.14 | -0.17 | 0.45 |
|  | Top quintile -- national wealth score | -0.11 | -0.47 | 0.26 | 0.33 | -0.01 | 0.67 |
|  | Number of pharmacies within 3 km | 0.01 | -0.01 | 0.03 | -0.01 | -0.03 | 0.01 |
|  | Number of public facilities within 3 km | -0.11 | -0.22 | 0.01 | 0.09 | -0.01 | 0.18 |
|  | Number of outreach facilities within 3 km | -0.01 | -0.18 | 0.16 | 0.11 | -0.03 | 0.25 |
|  | Mean number of methods at facilities within 3 km | 0.15 | -0.44 | 0.74 | 0.09 | -0.28 | 0.46 |
|  | Constant | -0.56 | -2.77 | 1.65 | -1.03 | -2.44 | 0.38 |
| Long acting methods, linked to a facility | |  |  |  |  |  |  |
|  | Age 20-24 (ref 18-19) | -0.35 | -1.17 | 0.48 | N/A | N/A | N/A |
|  | 30-34 (ref 25-29) | N/A | N/A | N/A | -0.64 | -1.14 | -0.15 |
|  | 35-39 (ref 25-29) | N/A | N/A | N/A | -1.29 | -1.87 | -0.71 |
|  | 40-44 (ref 25-29) | N/A | N/A | N/A | -1.64 | -2.36 | -0.91 |
|  | 45-49 (ref 25-29 | N/A | N/A | N/A | -2.58 | -3.59 | -1.58 |
|  | No education | -16.08 | -1717.52 | 1685.37 | -0.89 | -1.65 | -0.13 |
|  | Muslim religion | -2.36 | -3.73 | -0.99 | -1.06 | -1.78 | -0.34 |
|  | Parity 1 | 4.64 | 2.58 | 6.71 | 3.61 | 1.56 | 5.67 |
|  | Parity 2 | 5.77 | 3.57 | 7.96 | 4.49 | 2.45 | 6.53 |
|  | Parity 2 or more | 6.08 | 3.72 | 8.45 | 4.78 | 2.74 | 6.81 |
|  | Married/in union | 0.95 | 0.19 | 1.71 | 1.12 | 0.59 | 1.65 |
|  | Top quintile -- national wealth score | -0.64 | -1.36 | 0.08 | 0.10 | -0.36 | 0.56 |
|  | Number of pharmacies within 3 km | 0.01 | -0.03 | 0.05 | -0.05 | -0.07 | -0.02 |
|  | Number of public facilities within 3 km | -0.10 | -0.33 | 0.13 | 0.15 | 0.01 | 0.29 |
|  | Number of outreach facilities within 3 km | 0.04 | -0.25 | 0.33 | 0.37 | 0.18 | 0.57 |
|  | Mean number of methods at facilities within 3 km | 0.85 | 0.05 | 1.66 | -0.06 | -0.55 | 0.43 |
|  | Constant | -8.25 | -11.89 | -4.61 | -5.50 | -8.16 | -2.84 |
| Short acting methods, linked to a facility | |  |  |  |  |  |  |
|  | Age 20-24 (ref 18-19) | 1.22 | 0.57 | 1.87 | N/A | N/A | N/A |
|  | 30-34 (ref 25-29) | N/A | N/A | N/A | -0.58 | -0.95 | -0.20 |
|  | 35-39 (ref 25-29) | N/A | N/A | N/A | -1.14 | -1.56 | -0.73 |
|  | 40-44 (ref 25-29) | N/A | N/A | N/A | -1.48 | -1.97 | -0.98 |
|  | 45-49 (ref 25-29 | N/A | N/A | N/A | -2.36 | -2.96 | -1.75 |
|  | No education | -4.42 | -6.43 | -2.42 | -2.09 | -2.70 | -1.49 |
|  | Muslim religion | -1.42 | -2.18 | -0.67 | -0.42 | -0.89 | 0.05 |
|  | Parity 1 | 1.18 | 0.66 | 1.69 | 1.40 | 0.86 | 1.94 |
|  | Parity 2 | 1.62 | 0.79 | 2.46 | 1.98 | 1.44 | 2.52 |
|  | Parity 2 or more | 1.14 | -0.14 | 2.42 | 2.36 | 1.81 | 2.91 |
|  | Married/in union | 0.83 | 0.35 | 1.32 | 0.70 | 0.38 | 1.02 |
|  | Top quintile -- national wealth score | -0.17 | -0.61 | 0.26 | 0.29 | -0.04 | 0.63 |
|  | Number of pharmacies within 3 km | 0.01 | -0.02 | 0.03 | -0.02 | -0.04 | 0.00 |
|  | Number of public facilities within 3 km | -0.07 | -0.21 | 0.07 | 0.11 | 0.01 | 0.21 |
|  | Number of outreach facilities within 3 km | 0.01 | -0.19 | 0.20 | 0.16 | 0.02 | 0.29 |
|  | Mean number of methods at facilities within 3 km | 1.20 | 0.46 | 1.94 | 0.37 | 0.01 | 0.72 |
|  | Constant | -6.79 | -9.71 | -3.87 | -3.09 | -4.48 | -1.70 |

**Supplemental Materials 2: Results of multnomial logistic regression predicting method use among women aged 18-24 and aged 25-49 in the CM4FP household survey in Uganda, 2019-2020**

In this section, we present the results of the multinomial logit models estimating determinants of contraceptive method choice among matched users, unmatched users, and non-users in Uganda. As above, we used non-use as the base category, so the coefficients can be interpreted as the effect of each explanatory variable on the log odds of being in one of the other method choice categories relative to being a non-user. The results for the younger sample in Uganda are largely driven by the negative effects of being in the 18 to 19 age category and the positive effects for the set of three parity dummy variables. Because of the extremely large number of private outlets, we used a 2 KM radius for the outlet related access variables. However, none of these variables had much explanatory power across all the comparisons. As expected because of the much larger sample size, there were more significant effects for the older age group. In addition to the expected results for the age and parity dummies, the no education dummy was significant in several comparisons and in the hypothesized negative direction while marital status had a positive effect and was significant in most cases. Finally, the average number of methods available within 2 KM for the long acting not linked and the short acting linked comparisons were positive and significant in the older age sample.

**Table S2: Multnomial logistic regression predicting method use among women aged 18-24 and aged 25-49 in CM4FP household survey in Uganda, 2019-2020**

|  |  | **Age 18-24** |  | | | | | | **Age 25-49** | | |  | | |  | | | |
| --- | --- | --- | --- | --- | --- | --- | --- | --- | --- | --- | --- | --- | --- | --- | --- | --- | --- | --- |
|  |  | **Coefficient** | **95%CI** | | | | | | **Coefficient** | | | **95%CI** | | |  | | | |
| None (base outcome) | | |  | |  | | | |  | | |  | | | | | | |
| Traditional methods | | |  |  | |  | | |  | | |  | | |  |  |  |  |
|  | Age 18-19 | -0.99 | -1.57 | | -0.40 | | | |  | | |  | | |  | | | |
|  | 30-34 (ref 25-29) | N/A | N/A | | N/A | | | | -0.39 | | | -0.77 | | | -0.02 | | | |
|  | 35-39 (ref 25-29) | N/A | N/A | | N/A | | | | -0.67 | | | -1.10 | | | -0.23 | | | |
|  | 40-44 (ref 25-29) | N/A | N/A | | N/A | | | | -0.80 | | | -1.30 | | | -0.29 | | | |
|  | 45-49 (ref 25-29 | N/A | N/A | | N/A | | | | -1.99 | | | -2.64 | | | -1.34 | | | |
|  | No education | N/A | N/A | | N/A | | | | -0.45 | | | -1.06 | | | 0.16 | | | |
|  | Muslim | 0.04 | -0.57 | | 0.65 | | | | -0.22 | | | -0.65 | | | 0.20 | | | |
|  | Parity 1 | 0.62 | 0.09 | | 1.15 | | | | 0.26 | | | -0.31 | | | 0.82 | | | |
|  | Parity 2 | 0.78 | 0.07 | | 1.50 | | | | -0.30 | | | -0.86 | | | 0.27 | | | |
|  | Parity 3 or more | -0.54 | -1.53 | | 0.44 | | | | 0.49 | | | -0.05 | | | 1.02 | | | |
|  | Married/in union | 0.50 | 0.00 | | 1.00 | | | | 1.43 | | | 1.06 | | | 1.81 | | | |
|  | Top quintile -- national wealth score | -0.40 | -0.90 | | 0.10 | | | | -0.15 | | | -0.50 | | | 0.21 | | | |
|  | Number of pharmacies within 2 km | -0.03 | -0.11 | | 0.04 | | | | 0.00 | | | -0.05 | | | 0.04 | | | |
|  | Number of public/private facilities within 2 km | -0.08 | -0.33 | | 0.18 | | | | -0.07 | | | -0.24 | | | 0.10 | | | |
|  | Number of facilities with outreach within 2 km | 0.01 | -0.10 | | 0.12 | | | | -0.01 | | | -0.09 | | | 0.07 | | | |
|  | Mean number of methods at facilities within 2 km | -0.05 | -0.35 | | 0.25 | | | | -0.10 | | | -0.29 | | | 0.09 | | | |
|  | Constant | -0.25 | -1.14 | | 0.64 | | | | -0.67 | | | -1.35 | | | 0.01 | | | |
| Long acting methods, not linked to a facility | | | | | | | |  |  |  | |  |  |  |  |  |  |  |
|  | Age 18-19 | -1.36 | -2.36 | | -0.36 | | | |  | | |  | | |  | | | |
|  | 30-34 (ref 25-29) | N/A | N/A | | N/A | | | | -0.42 | | | -0.83 | | | 0.00 | | | |
|  | 35-39 (ref 25-29) | N/A | N/A | | N/A | | | | -1.21 | | | -1.75 | | | -0.67 | | | |
|  | 40-44 (ref 25-29) | N/A | N/A | | N/A | | | | -1.12 | | | -1.73 | | | -0.52 | | | |
|  | 45-49 (ref 25-29 | N/A | N/A | | N/A | | | | -2.09 | | | -2.87 | | | -1.30 | | | |
|  | No education | N/A | N/A | | N/A | | | | -1.01 | | | -1.98 | | | -0.05 | | | |
|  | Muslim | -0.64 | -1.47 | | 0.19 | | | | -0.27 | | | -0.77 | | | 0.22 | | | |
|  | Parity 1 | 2.83 | 1.97 | | 3.68 | | | | 0.81 | | | -0.02 | | | 1.63 | | | |
|  | Parity 2 | 3.40 | 2.41 | | 4.40 | | | | 0.95 | | | 0.15 | | | 1.74 | | | |
|  | Parity 3 or more | 1.85 | 0.54 | | 3.16 | | | | 1.73 | | | 0.95 | | | 2.52 | | | |
|  | Married/in union | -0.28 | -0.90 | | 0.34 | | | | 1.06 | | | 0.63 | | | 1.48 | | | |
|  | Top quintile -- national wealth score | 0.68 | -0.01 | | 1.38 | | | | 0.36 | | | -0.07 | | | 0.80 | | | |
|  | Number of pharmacies within 2 km | -0.05 | -0.14 | | 0.04 | | | | -0.02 | | | -0.07 | | | 0.04 | | | |
|  | Number of public/private facilities within 2 km | 0.22 | -0.09 | | 0.54 | | | | 0.03 | | | -0.16 | | | 0.22 | | | |
|  | Number of facilities with outreach within 2 km | 0.04 | -0.11 | | 0.19 | | | | -0.09 | | | -0.18 | | | 0.00 | | | |
|  | Mean number of methods at facilities within 2 km | -0.12 | -0.55 | | 0.30 | | | | 0.28 | | | 0.01 | | | 0.55 | | | |
|  | Constant | -2.87 | -4.23 | | -1.51 | | | | -3.00 | | | -4.02 | | | -1.97 | | | |
| Short acting methods, not linked to a facility | | | | | | | |  |  |  | |  |  |  |  |  |  |  |
|  | Age 18-19 | -0.67 | -1.09 | | -0.25 | | | |  | | |  | | |  | | | |
|  | 30-34 (ref 25-29) | N/A | N/A | | N/A | | | | -0.30 | | | -0.65 | | | 0.05 | | | |
|  | 35-39 (ref 25-29) | N/A | N/A | | N/A | | | | -0.46 | | | -0.88 | | | -0.05 | | | |
|  | 40-44 (ref 25-29) | N/A | N/A | | N/A | | | | -1.00 | | | -1.54 | | | -0.46 | | | |
|  | 45-49 (ref 25-29 | N/A | N/A | | N/A | | | | -1.65 | | | -2.31 | | | -0.99 | | | |
|  | No education | N/A | N/A | | N/A | | | | -1.04 | | | -1.82 | | | -0.26 | | | |
|  | Muslim | -0.32 | -0.82 | | 0.17 | | | | -0.36 | | | -0.75 | | | 0.04 | | | |
|  | Parity 1 | 0.72 | 0.29 | | 1.15 | | | | 0.19 | | | -0.28 | | | 0.65 | | | |
|  | Parity 2 | 0.47 | -0.19 | | 1.12 | | | | 0.04 | | | -0.42 | | | 0.49 | | | |
|  | Parity 3 or more | -0.21 | -1.04 | | 0.62 | | | | 0.10 | | | -0.37 | | | 0.56 | | | |
|  | Married/in union | -0.09 | -0.48 | | 0.29 | | | | 0.42 | | | 0.12 | | | 0.71 | | | |
|  | Top quintile -- national wealth score | 0.00 | -0.39 | | 0.39 | | | | 0.16 | | | -0.19 | | | 0.50 | | | |
|  | Number of pharmacies within 2 km | 0.01 | -0.05 | | 0.07 | | | | 0.04 | | | -0.01 | | | 0.09 | | | |
|  | Number of public/private facilities within 2 km | -0.06 | -0.25 | | 0.13 | | | | 0.06 | | | -0.09 | | | 0.21 | | | |
|  | Number of facilities with outreach within 2 km | -0.01 | -0.10 | | 0.08 | | | | 0.01 | | | -0.06 | | | 0.08 | | | |
|  | Mean number of methods at facilities within 2 km | 0.02 | -0.26 | | 0.31 | | | | -0.09 | | | -0.33 | | | 0.14 | | | |
|  | Constant | -0.09 | -0.91 | | 0.74 | | | | -0.77 | | | -1.49 | | | -0.06 | | | |
| Long acting methods, linked to a facility | | | |  | | |  | |  | |  | |  | | |  | |  |
|  | Age 18-19 | -0.55 | -1.60 | | 0.50 | | | |  | | |  | | |  | | | |
|  | 30-34 (ref 25-29) | N/A | N/A | | N/A | | | | -0.85 | | | -1.46 | | | -0.25 | | | |
|  | 35-39 (ref 25-29) | N/A | N/A | | N/A | | | | -2.47 | | | -3.56 | | | -1.38 | | | |
|  | 40-44 (ref 25-29) | N/A | N/A | | N/A | | | | -2.78 | | | -4.26 | | | -1.30 | | | |
|  | 45-49 (ref 25-29 | N/A | N/A | | N/A | | | | -2.97 | | | -4.45 | | | -1.49 | | | |
|  | No education | N/A | N/A | | N/A | | | | -14.44 | | | -1331.61 | | | 1302.74 | | | |
|  | Muslim | -0.58 | -1.57 | | 0.41 | | | | 0.26 | | | -0.46 | | | 0.97 | | | |
|  | Parity 1 | 4.41 | 2.37 | | 6.45 | | | | 14.41 | | | -922.00 | | | 950.81 | | | |
|  | Parity 2 | 4.28 | 2.12 | | 6.44 | | | | 14.63 | | | -921.77 | | | 951.04 | | | |
|  | Parity 3 or more | 3.57 | 1.25 | | 5.89 | | | | 15.53 | | | -920.88 | | | 951.93 | | | |
|  | Married/in union | 0.31 | -0.53 | | 1.15 | | | | 0.73 | | | 0.07 | | | 1.40 | | | |
|  | Top quintile -- national wealth score | 0.11 | -0.70 | | 0.93 | | | | 0.06 | | | -0.59 | | | 0.71 | | | |
|  | Number of pharmacies within 2 km | -0.06 | -0.18 | | 0.05 | | | | -0.06 | | | -0.15 | | | 0.04 | | | |
|  | Number of public/private facilities within 2 km | 0.00 | -0.40 | | 0.40 | | | | 0.24 | | | -0.08 | | | 0.56 | | | |
|  | Number of facilities with outreach within 2 km | 0.06 | -0.12 | | 0.24 | | | | -0.03 | | | -0.18 | | | 0.13 | | | |
|  | Mean number of methods at facilities within 2 km | 0.27 | -0.32 | | 0.86 | | | | -0.03 | | | -0.37 | | | 0.32 | | | |
|  | Constant | -5.92 | -8.53 | | -3.31 | | | | -16.30 | | | -952.70 | | | 920.11 | | | |
| Short acting methods, linked to a facility | | | |  | | | |  |  | | |  | |  | | |  | |
|  | Age 18-19 | -0.98 | -1.60 | | -0.37 | | | |  | | |  | | |  | | | |
|  | 30-34 (ref 25-29) | N/A | N/A | | N/A | | | | -0.61 | | | -0.98 | | | -0.25 | | | |
|  | 35-39 (ref 25-29) | N/A | N/A | | N/A | | | | -0.85 | | | -1.28 | | | -0.42 | | | |
|  | 40-44 (ref 25-29) | N/A | N/A | | N/A | | | | -1.38 | | | -1.93 | | | -0.82 | | | |
|  | 45-49 (ref 25-29 | N/A | N/A | | N/A | | | | -2.70 | | | -3.54 | | | -1.87 | | | |
|  | No education | N/A | N/A | | N/A | | | | -0.47 | | | -1.12 | | | 0.18 | | | |
|  | Muslim | -0.05 | -0.63 | | 0.52 | | | | 0.23 | | | -0.14 | | | 0.61 | | | |
|  | Parity 1 | 1.27 | 0.75 | | 1.79 | | | | 0.77 | | | 0.21 | | | 1.33 | | | |
|  | Parity 2 | 1.34 | 0.62 | | 2.06 | | | | 0.82 | | | 0.28 | | | 1.37 | | | |
|  | Parity 3 or more | 0.46 | -0.51 | | 1.42 | | | | 1.14 | | | 0.59 | | | 1.69 | | | |
|  | Married/in union | 0.03 | -0.45 | | 0.51 | | | | 0.23 | | | -0.08 | | | 0.53 | | | |
|  | Top quintile -- national wealth score | 0.30 | -0.21 | | 0.81 | | | | -0.24 | | | -0.58 | | | 0.09 | | | |
|  | Number of pharmacies within 2 km | -0.01 | -0.08 | | 0.06 | | | | 0.00 | | | -0.05 | | | 0.05 | | | |
|  | Number of public/private facilities within 2 km | -0.01 | -0.25 | | 0.22 | | | | -0.04 | | | -0.20 | | | 0.12 | | | |
|  | Number of facilities with outreach within 2 km | -0.02 | -0.13 | | 0.08 | | | | -0.06 | | | -0.13 | | | 0.02 | | | |
|  | Mean number of methods at facilities within 2 km | 0.19 | -0.20 | | 0.59 | | | | 0.53 | | | 0.24 | | | 0.82 | | | |
|  | Constant | -1.77 | -2.95 | | -0.59 | | | | -2.31 | | | -3.27 | | | -1.35 | | | |

**Supplementary Table S3: Mixed logit with selection correction predicting facility choice among women aged 18-24 and aged 25-49 in CM4FP household survey in Kenya, 2019-2020**

|  |  |  | |  |  |  |
| --- | --- | --- | --- | --- | --- | --- |
|  | **Age 18-24** | | | **Age 25-49** |  |  |
|  | **Coefficient** | **95% CI** | | **Coefficient** | **95%CI** |  |
| Mean |  |  |  |  |  |  |
| Open 7 days/week | 0.48 | -0.04 | 1.01 | 0.08 | -0.21 | 0.37 |
| Host outreach training | 0.30 | -0.16 | 0.76 | 0.73 | 0.47 | 0.98 |
| Provider training | 0.73 | 0.33 | 1.13 | 0.79 | 0.55 | 1.02 |
| Pharmacy | 0.94 | 0.32 | 1.55 | 0.07 | -0.31 | 0.44 |
| Combined distance | -1.85 | -2.47 | -1.22 | -1.80 | -2.13 | -1.46 |
| Number of methods offered | 0.23 | 0.04 | 0.41 | 0.24 | 0.13 | 0.34 |
| Public facility | 3.35 | 0.34 | 6.37 | 3.26 | 1.92 | 4.60 |
| SD |  |  |  |  |  |  |
| Combined distance | 1.19 | 0.61 | 1.77 | 1.00 | 0.75 | 1.26 |
| Number of methods offered | 0.55 | 0.27 | 0.84 | 0.46 | 0.30 | 0.62 |
| Public facility | -12.44 | -21.71 | -3.17 | 6.17 | 1.20 | 11.13 |

**Supplementary Table S4: Mixed logit with selection correction predicting facility choice among women aged 18-24 and aged 25-49 in CM4FP household survey in Uganda, 2019-2020**

|  | Age 18-24 |  | | Age 25-49 |  |  |
| --- | --- | --- | --- | --- | --- | --- |
|  | Coefficient | 95% CI | | Coefficient | 95% CI |  |
| Mean |  |  |  |  |  |  |
| Stock out of injectables | -0.72 | -1.47 | 0.02 | -0.48 | -0.89 | -0.06 |
| Provider training | 0.35 | -0.08 | 0.77 | -0.24 | -0.54 | 0.06 |
| FP signage | 0.28 | -0.14 | 0.70 | 0.53 | 0.25 | 0.81 |
| Public & public/private | 2.72 | 2.16 | 3.28 | 2.37 | 2.02 | 2.72 |
| Pharmacy | 1.72 | 1.22 | 2.23 | 1.60 | 1.26 | 1.94 |
| Combined distance | -1.36 | -1.99 | -0.74 | -1.64 | -2.00 | -1.27 |
| Number of methods offered | 0.40 | 0.22 | 0.57 | 0.52 | 0.41 | 0.64 |
| Open 7 days/week | 0.79 | -0.84 | 2.41 | 0.33 | -0.83 | 1.48 |
| SD |  |  |  |  |  |  |
| Combined distance | 0.92 | 0.34 | 1.50 | 1.08 | 0.74 | 1.42 |
| Number of methods offered | 0.32 | -0.21 | 0.84 | -0.18 | -0.43 | 0.08 |
| Open 7 days/week | 2.19 | -0.40 | 4.78 | 2.61 | 0.35 | 4.87 |
